# Scrub Typhus and the Misconception of Doxycycline Resistance

**DOI:** 10.1093/cid/ciz972

**Published:** 2019-10-01

**Authors:** Tri Wangrangsimakul, Weerawat Phuklia, Paul N Newton, Allen L Richards, Nicholas P J Day

**Affiliations:** 1 Mahidol -Oxford Tropical Medicine Research Unit, Faculty of Tropical Medicine, Mahidol University, Bangkok, Thailand; 2 Centre for Tropical Medicine and Global Health, Nuffield Department of Clinical Medicine, University of Oxford, Oxford, United Kingdom; 3 Lao-Oxford-Mahosot Hospital–Wellcome Trust Research Unit, Microbiology Laboratory, Mahosot Hospital, Vientiane, Lao People’s Democratic Republic; 4 Department of Preventive Medicine and Biostatistics, Uniformed Services University of the Health Sciences, Bethesda, Maryland, USA

**Keywords:** scrub typhus, *Orientia tsutsugamushi*, doxycycline, resistance, treatment outcomes

## Abstract

Scrub typhus, a neglected infectious disease caused by the obligate intracellular bacterium *Orientia tsutsugamushi*, is a major cause of fever across the Asia Pacific region with more than a billion people at risk. Treatment with antibiotics such as doxycycline or chloramphenicol is effective for the majority of patients. In the 1990s, reports from northern Thailand raised a troubling observation; some scrub typhus patients responded poorly to doxycycline, which investigators attributed to doxycycline resistance. Despite the controversial nature of these reports, independent verification was neglected, with subsequent studies speculating on the role of doxycycline resistance in contributing to failure of treatment or prophylaxis. In this review, we have outlined the evidence for drug-resistant *Orientia tsutsugamushi*, assessed the evidence for doxycycline resistance, and highlight more recent findings unsupportive of doxycycline resistance. We conclude that doxycycline resistance is a misconception, with treatment outcome likely to be determined by other bacterial, host, and pharmacological factors.

## Teaser

Scrub typhus is a major cause of fever across the Asia Pacific region. Resistance to doxycycline, the main antibiotic used for treatment, was described in the 1990s. Assessment of historical evidence and recent reports suggest this finding was a misconception.

Scrub typhus, a mite-borne neglected infectious disease caused by the obligate gram-negative bacterium *Orientia tsutsugamushi*, is a major cause of acute undifferentiated fever across large regions of the Asia Pacific, with more than a billion people at risk of contracting the disease. Recent reviews have estimated the median untreated and treated mortalities at 6.0% and 1.4%, respectively [1, 2]. Prior to the discovery of chloramphenicol (Chloromycetin) in 1948 as an effective treatment for scrub typhus, the disease was feared, especially by troops during World War II in whom significant morbidity and mortality were reported [3, 4]. Further progress came with the discovery of the tetracyclines as a safer and more effective alternative, with doxycycline established as the standard treatment [5, 6]. The response to therapy in the majority was dramatic, giving rise to the dictum that failure to respond to treatment with a tetracycline antibiotic within 48 hours is suggestive of an alternative diagnosis, such as typhoid [7]. However, in the 1990s, clinicians in northern Thailand informed US Army researchers of a troubling observation; despite appropriate antibiotics, some patients with scrub typhus failed to respond to treatment, with fatal consequences in a few.

## EVIDENCE FOR DRUG-RESISTANT *O. TSUTSUGAMUSHI*

In response to these reports, a small clinical trial was carried out involving patients with scrub typhus in Thailand—12 from Chiang Rai, Chiang Rai Province, and 7 from Mae Sot, Tak Province—and reported in 1996 [8]. Fever cleared within 3 days in all 7 patients from Mae Sot whereas only 5 of 12 patients from Chiang Rai defervesced in the same time period. All patients were treated with a 7-day course of doxycycline and had scrub typhus diagnosis confirmed by serology using admission blood samples. *Orientia tsutsugamushi* isolates were cultivated by injecting blood from patients into the peritoneal cavity of mice. Susceptibility testing was performed on 3 selected clinical isolates and a reference Karp strain using a mouse survivability model and cell culture. The results appeared to show that 1 *O. tsutsugamushi* isolate, labeled AFC-3, was resistant to doxycycline with a minimum inhibitory concentration (MIC) of >4 µg/mL whereas another isolate, labeled AFC-27, was at least partially resistant to doxycycline [8]. Results from an *O. tsutsugamushi* in vitro susceptibility study were published around the same period [9]. In this study, an *O. tsutsugamushi* isolate, AFSC-4, cultured from a patient with delayed response to antibiotic treatment from western Thailand in 1990, was found to be less susceptible to doxycycline than the reference Karp strain whereas azithromycin appeared effective for both strains [9]. The doxycycline MIC for AFSC-4 was estimated to be at least 0.25–0.5 µg/mL whereas for Karp, it was 0.0625 µg/mL. It was concluded that AFSC-4 was resistant to doxycycline.

More recent clinical studies from southern India, South Korea, and northeastern Thailand reporting severe intractable disease and some deaths have speculated on the role of doxycycline resistance [10–12]. Failure of prophylaxis in military personnel in Southeast Asia and Australia has also been reported with the presumption that doxycycline resistance contributed [13, 14]. However, only the Australian study sought to clarify the findings. Culture for *O. tsutsugamushi* and susceptibility testing was performed and doxycycline resistance was ruled out in 1 patient. A third *O. tsutsugamushi* isolate, AFSC-7, cultured in 1990, was also reported as doxycycline insensitive, but the original characterization and antibiotic susceptibility testing (AST) have not been published [15]. There have been additional *O. tsutsugamushi* isolates, cultured from patients from northern Thailand as part of a larger clinical trial in the 1990s with delayed responses to therapy and studied by the US military, but the detailed methodology and results remain unpublished, with only a short summary available in a recent review [16].

Evidence for innate antibiotic resistance in *O. tsutsugamushi* has been described. In silico analysis of multiple reference and clinical *O. tsutsugamushi* strains have revealed a Ser83Leu *gyrA* mutation in all studied strains, suggesting intrinsic fluoroquinolone resistance [17, 18]. β-Lactams, sulphonamides, and aminoglycosides have been shown to be ineffective in humans, animals, and in vitro [19–21]. The natural resistance of *O. tsutsugamushi* to β-lactams have previously been attributed to the absence of peptidoglycan, but recent reports suggest that a peptidoglycan remnant is present [22].

## ASSESSING THE EVIDENCE FOR DOXYCYCLINE RESISTANCE

Unsurprisingly, the initial reports were controversial at the time and remain so today. If doxycycline resistance in *O. tsutsugamushi* is not innate and is acquired, where did the antibiotic selective pressure originate [4]? The larval stage of the vector trombiculid mite is thought to feed only once on vertebrate hosts prior to continuing their development in the soil environment [23]. Experimental studies to infect larval stages of the mites through feeding on infected rodents have rarely succeeded [24]. Antibiotic exposure through animal feeds or the environment has also been postulated as drivers of resistance, but these conditions are not unique to northern Thailand [4, 8]. Further explanations have speculated on spontaneous mutation, without selective pressure, leading to acquired doxycycline resistance, based on the high rates of homologous recombination of *O. tsutsugamushi* or the presence of innately doxycycline-resistant strains hitherto undiscovered [16, 25]. However, despite ongoing references to doxycycline resistance in *O. tsutsugamushi*, the clinical study from Chiang Rai (AFC-3 isolate) and the in vitro susceptibility study (AFSC-4 isolate) remain the only studies published providing any objective evidence of potential resistance.

It is important to recognize that there remains no internationally agreed-upon reference standard for AST of *O. tsutsugamushi*. The procedures for in vitro isolation of *O. tsutsugamushi* in mammalian cell lines (eg, L929 mouse fibroblast cells) and AST have been reviewed elsewhere [16]. On closer scrutiny, the AST methodology used in both of the original doxycycline resistance studies had significant shortcomings. In the Chiang Rai study, L929 cells were infected with clinical *O. tsutsugamushi* isolates and compared to the reference Karp strain [8]. Infected cells were incubated with media containing no antibiotics, 4 µg/mL and 16 µg/mL of doxycycline, or 8 µg/mL and 32 µg/mL of chloramphenicol. However, after only 30 hours’ incubation, the cells were removed, fixed in methanol, Giemsa stained, and examined for infectivity by microscopy. In essence, a short incubation period and high antibiotic concentrations, similar to AST for extracellular bacteria, were utilized for the obligate intracellular *O. tsutsugamushi*, which has vastly different growth dynamics. At that time, AST in related *Rickettsia* species had been performed, initially using an incubation period of at least 4 days, with subsequent studies incubating between 5 and 10 days depending on the specific assay [26, 27].

Similarly, the in vitro susceptibility study involving *O. tsutsugamushi* AFSC-4 isolate and the reference Karp strain utilized L929 cells for culture [9]. A greater range of antibiotic concentrations (doxycycline and azithromycin) was investigated, which included low concentrations and incubation for 3 days. Cells were examined for infectivity using microscopy and flow cytometry. The doxycycline MIC for AFSC-4 was higher than for Karp (at least 0.25–0.5 µg/mL vs 0.0625 µg/mL), whereas azithromycin was found to be effective for both isolates at similar MICs (0.0156 µg/mL for AFSC-4 and 0.0078 µg/mL for Karp). It was concluded that the AFSC-4 isolate was doxycycline resistant despite the plasma doxycycline concentrations achieved in humans at standard doses far exceeding 0.5 µg/mL [8, 28]. Screening of the *O. tsutsugamushi* isolates using extremely high concentrations of doxycycline (16 µg/mL) and azithromycin (8 µg/mL) was also performed [9]. After 3 days’ incubation, a significant number of cells remained infected for both isolates with both antibiotics despite the high doses used, suggesting that the incubation period may be suboptimal.

Although the growth cycle–dependent pharmacokinetic-pharmacodynamic relationships of antibiotics in *O. tsutsugamushi* have not been reported, this has been investigated in vitro in chlamydiae, which are closely related obligate intracellular bacteria [29]. Antibiotic treatment in the extracellular phase had minimal effect on infectivity with susceptibility maximal in the intracellular, metabolically active, replicative phase. In obligate intracellular bacteria, AST is complex and dependent on the characteristics of the pathogen growth cycle along with the pharmacokinetics, pharmacodynamics, and mechanism of action of the tested antibiotics. For *O. tsutsugamushi*, an AST assay with an incubation period that allows peak bacterial concentrations to be reached, for all extracellular-phase bacteria to transition to the replicative intracellular phase, and that allows sufficient time for the antibiotics to concentrate within the intracellular space and to exert their effects is necessary.

**Figure 1.**
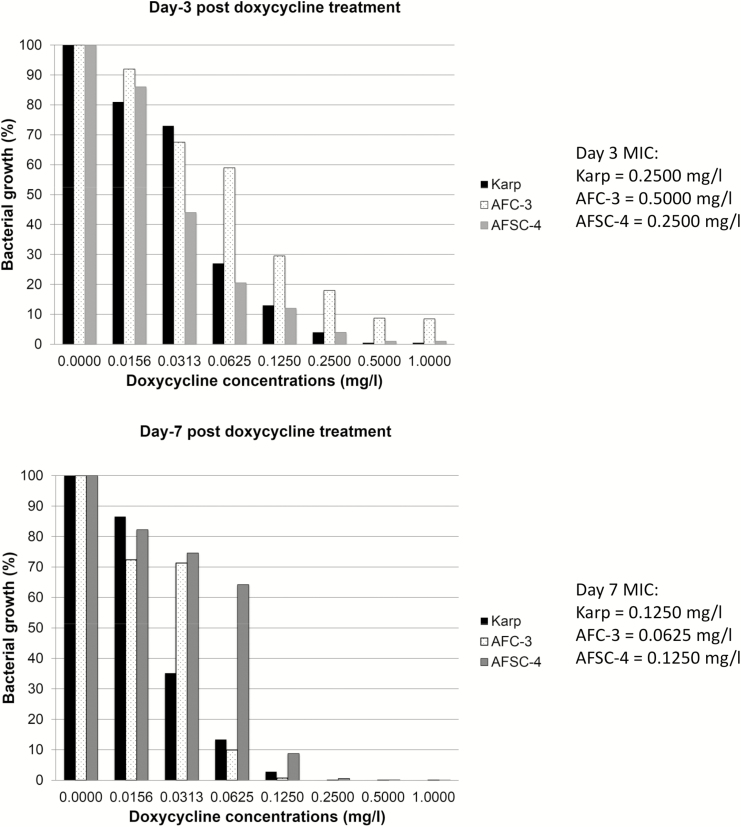
Doxycycline susceptibility testing of *Orientia tsutsugamushi* Karp, AFC-3, and AFSC-4 strains at 3 days vs 7 days of incubation posttreatment. MIC (minimum inhibitory concentration) represents drug concentrations that inhibit >90% of bacterial growth [Phuklia W, unpublished data, 2018].

Another potential weakness of previous AST assays was a reliance on cell counts by microscopy as a subjective measure of bacterial growth and replication. An AST method based on determining the *O. tsutsugamushi* load by quantitative polymerase chain reaction (PCR) has recently been developed [30]. The optimal incubation period, based on the growth kinetics of 5 reference strains (Karp, Kato, Gilliam, UT76, and TA763) and the 2 putatively doxycycline-resistant strains (AFC-3 and AFSC-4), was determined to be from 7 to 10 days, with 10 days chosen as the single cell-harvesting time-point. Doxycycline MIC_90_ values (drug concentration that inhibits >90% of bacterial growth) for Karp, AFC-3, and AFSC-4 were compared at day 3 and day 7 of incubation (Figure 1). The MIC_90_ values were lower for all tested *O. tsutsugamushi* isolates at the longer period of incubation. However, the in vitro AST assays described are unable to differentiate live and dead bacteria. In future, additional steps including RNA PCR, subcultures, or mouse inoculation may be considered to assess the viability of detectable bacteria following AST.

## EVIDENCE NOT SUPPORTING DOXYCYCLINE RESISTANCE

Despite the controversial nature of the reports from the 1990s, independent verification of the findings was surprisingly neglected. A relative lack of access to AFC-3 and AFSC-4 isolates and difficulties performing AST and determining an MIC for *O. tsutsugamushi*, classed as a risk group 3 pathogen, contributed [Blacksell SD, et al., Unpublished work, 2019]. It is only recently that AST of AFC-3 and AFSC-4 isolates were reexamined by 2 independent groups. In the first study, AFC-3, AFSC-4, Karp, and another isolate from the original Chiang Rai study, AFC-1, were tested using the methodology from the original AFSC-4 report [16]. Incubation was for 3 days and bacterial growth was assessed using microscopy to measure the number of orientiae per 100 L929 cells. Growth was inhibited at 0.1 µg/mL of doxycycline in all 4 strains, not supporting doxycycline resistance [16].

The second report, part of the *O. tsutsugamushi* AST assay development study utilizing 5 reference strains (Karp, Kato, Gilliam, UT76, and TA763) and AFC-3 and AFSC-4 isolates provided further evidence against the presence of doxycycline resistance [30]. The AST assay used an incubation period of 10 days and trypsinization for cell harvesting. Quantitative PCR targeting the 47-kDa *htra* gene was used to assess bacterial growth. The concentrations of azithromycin, doxycycline, and chloramphenicol found to inhibit >90% of bacterial growth were used as the MIC_90_. The doxycycline MIC_90_ for AFC-3 and AFSC-4 isolates (0.125 mg/L and 0.250 mg/L, respectively) were comparable to the other 5 reference strains, albeit slightly higher, and remained well below the drug levels achieved in vivo in human plasma [30]. In addition, a further 51 *O. tsutsugamushi* isolates from Thai and Lao patients underwent AST to determine antibiotic screening concentrations for azithromycin, doxycycline, and chloramphenicol. All MIC_90_ results suggested that the isolates were susceptible to all 3 antibiotics.

Furthermore, an *O. tsutsugamushi* isolate was cultured from admission blood samples of a pediatric patient from Chiang Rai with prolonged fever clearance time (150 hours) as part of a clinical study [31]. AST of this isolate, using the novel quantitative PCR–based assay described above, revealed an MIC_90_ of 0.0625 mg/L for doxycycline, indicating that doxycycline resistance did not contribute to prolonged fever clearance time in this patient.

These studies suggest that doxycycline resistance is a misconception, predicated on AST assays with significant shortcomings. Mutations of AFC-3 and AFSC-4 isolates through multiple passages and long-term storage leading to loss of doxycycline-resistant phenotype is a possible explanation [32]. However, recent analysis of the genetic stability of *O. tsutsugamushi* prototype strains Gilliam, Karp, and Kato over time and ongoing work on the genomic stability of *O. tsutsugamushi* UT76 and TM4942 strains through multiple passages suggest this is unlikely to be the case [32] (J. Salje, personal communication, April 2019). The presence of undiscovered naturally doxycycline-resistant *O. tsutsugamushi* strains remains a theoretical possibility. If doxycycline resistance is not real, then other factors may explain why poor treatment outcomes are seen in northern Thailand and elsewhere.

## DETERMINANTS OF TREATMENT OUTCOME

The dictum that treatment of scrub typhus almost always results in the rapid resolution of fever and other symptoms is an oversimplification. Analysis of fever clearance time from prospective treatment trials reveals that prolonged defervescence occurs in most settings, suggesting that treatment outcome is a spectrum [33]. The determinants are likely to be multifactorial and encompass host, bacterial, and pharmacological factors. Delays in disease recognition, seeking medical care, and administration of effective antibiotics contribute and raise the likelihood of the infection becoming established and disseminated. Late presentation with severe disease and multiorgan failure leads to higher mortality despite the initiation of appropriate antibiotics [34]. Older age is also associated with higher mortality [2]. Although the sample size in the original Chiang Rai study was small, patients from Chiang Rai were ill for longer and were older than their counterparts from Mae Sot, potentially confounding the treatment outcome [8]. Preexisting immunity through prior infections can protect the individual or at least ameliorate clinical infection [35]. Humoral responses are particularly important for homologous protection (same strain) while cellular immunity is key for heterologous protection (cross-strain) [35–37]. However, immunity is transient with homologous protection lasting from 1 year to a few years, whereas heterologous protection rarely extends beyond a year [35, 36]. This is a major challenge in scrub typhus, allowing for repeated infections in at-risk individuals, and is compounded by the high degree of genotypic diversity within endemic regions [38].

Bacterial factors affecting treatment outcome, including the diversity of virulence of *O. tsutsugamushi* isolates, have been described [39]. The infectivity and growth rate of AFSC-4 have been compared with other reference and clinical *O. tsutsugamushi* isolates in South Korea [40]. ECV304 cells were inoculated with the studied isolates and the proportion of infected cells was assessed at 4, 24, 28, and 72 hours by immunofluorescence staining and microscopy. At 4 hours, AFSC-4 showed higher infectivity than all other tested *O. tsutsugamushi* strains. The higher proportion of infected cells for AFSC-4 compared to other tested strains was maintained to 72 hours, although the shape of the growth curve did not differ significantly [40]. Previous studies in mice have concluded that the *O. tsutsugamushi* growth rate may be the determining factor in pathogenicity [41]. However, the Korean study highlights infectivity rather than growth rate per se as the crucial virulence factor [40].

Further evidence of high infectivity of AFSC-4 and AFC-3 has recently been reported as part of the *O. tsutsugamushi* AST assay development study from Southeast Asia [30]. The doubling time for AFC-3 and AFSC-4 strains were observed at 9.50 and 10.53 hours, respectively, and were clearly shorter than the other 5 reference strains tested (range, 12.03–19.85 hours). Although growth rates in the first 3 days were not assessed, growth curve analysis from day 3 to day 12 postinfection showed that AFC-3 and AFSC-4 reached a set bacterial load faster than other tested strains [30]. This is clinically significant, as high *O. tsutsugamushi* load is associated with severe disease and treatment failure is much more likely in this scenario [42].

Pharmacological factors will also determine treatment outcome. There is growing realization of the risks of poor-quality antibiotics and the potential risk for bias in clinical trials [43]. Substandard formulation, in terms of inadequate doxycycline composition and bioavailability, would have reduced in vivo exposure of *O. tsutsugamushi* to doxycycline, leading to impaired clinical response. The ability of the drug to penetrate the intracellular compartment of *O. tsutsugamushi*–tropic cells, such as peripheral blood mononuclear cells and endothelial cells, is also crucial [44]. Doxycycline and azithromycin both interfere with bacterial protein synthesis and both will affect the active replicative intracellular phase of the *O. tsutsugamushi* growth cycle. Both drugs concentrate well in the intracellular space of white blood cells but to different degrees; azithromycin cellular concentrations can be >100 times higher than plasma or serum in healthy human volunteers, whereas doxycycline cellular concentrations may only reach around 10 times the values measured in the extracellular compartment [45–47]. In the initial study comparing the susceptibility of AFSC-4 and Karp strains to doxycycline and azithromycin, azithromycin may have appeared to be more effective than doxycycline due its greater ability to concentrate in the intracellular space, an effect amplified by a shortened culture incubation period [9]. Growing evidence for the efficacy of rifampicin in treating scrub typhus could be partially explained by the antibiotic’s ability to concentrate well within white blood cells [47]. There are suggestions that current doses of doxycycline may be suboptimal for the treatment of severe scrub typhus [48]. Previous trials comparing the efficacy of different antibiotics in treating scrub typhus are limited by their heterogeneity and the fact that the majority were underpowered [33]. The optimal choice of antibiotic for treatment remains unclear. Further pharmacological studies and high-quality clinical trials on the treatment of scrub typhus are urgently required.

## Conclusions

The evidence for doxycycline-resistant *O. tsutsugamushi* has always been limited. The controversial nature of these findings should have prompted further independent verification but this was neglected, creating uncertainty in the ensuing decades with doxycycline resistance being speculated upon as the cause of failure of treatment or prophylaxis. The 2 “doxycycline-resistant” *O. tsutsugamushi* strains (AFC-3 and AFSC-4) are now accessible and researchers have been able to study their growth characteristics and perform AST. The current evidence suggests that doxycycline resistance is a misconception. There are alternative valid explanations as to why AFC-3 and AFSC-4 strains differ from other studied strains and how these bacterial attributes, along with other host and pharmacological factors, contribute to variations in treatment outcome. Clinical trials embedding detailed analyses of these contributing factors are currently under way.

## Notes

***Acknowledgments.*** The authors thank Jeanne Salje for helpful discussions on the genomic stability of *Orientia tsutsugamushi*.

***Financial support.*** T. W., W. P., P. N. N., and N. P. J. D. are funded by the Wellcome Trust, United Kingdom, as part of the Mahidol-Oxford Tropical Medicine Research Unit Tropical Health Network institutional funding support.

***Potential conflicts of interest.*** The authors: No reported conflicts of interest. All authors have submitted the ICMJE Form for Disclosure of Potential Conflicts of Interest.
